# Imaging features of adrenal masses

**DOI:** 10.1186/s13244-019-0688-8

**Published:** 2019-01-25

**Authors:** Domenico Albano, Francesco Agnello, Federico Midiri, Giusy Pecoraro, Alberto Bruno, Pierpaolo Alongi, Patrizia Toia, Giuseppe Di Buono, Antonino Agrusa, Luca Maria Sconfienza, Salvatore Pardo, Ludovico La Grutta, Massimo Midiri, Massimo Galia

**Affiliations:** 1 Unità di Radiologia Diagnostica ed Interventistica, IRCCS Istituto Ortopedico Galeazzi, Via Riccardo Galeazzi 4, 20161 Milan, Italy; 2 Dipartimento di Biomedicina, Neuroscienze e Diagnostica Avanzata, Università degli studi di Palermo, Via del Vespro 127, 90127 Palermo, Italy; 3 Dipartimento di Discipline Chirurgiche Oncologiche e Stomatologiche, Università degli Studi di Palermo, Via Liborio Giuffrè 5, 90127 Palermo, Italy; 4 Department of General Surgery, Urgency and Organ Transplantation, University of Palermo, Via del Vespro 127, 90127 Palermo, Italy; 5 Department of Biomedical Sciences for Health, Università degli Studi di Milano, Via Festa del Perdono 7, 20122 Milan, Italy; 6 Department PROMISE, University of Palermo, Via del Vespro 127, 90127 Palermo, Italy

**Keywords:** Magnetic resonance imaging, Computed tomography, Adenoma, Adrenal, Chemical shift imaging

## Abstract

The widespread use of imaging examinations has increased the detection of incidental adrenal lesions, which are mostly benign and non-functioning adenomas. The differentiation of a benign from a malignant adrenal mass can be crucial especially in oncology patients since it would greatly affect treatment and prognosis. In this setting, imaging plays a key role in the detection and characterization of adrenal lesions, with several imaging tools which can be employed by radiologists. A thorough knowledge of the imaging features of adrenal masses is essential to better characterize these lesions, avoiding a misinterpretation of imaging findings, which frequently overlap between benign and malignant conditions, thus helping clinicians and surgeons in the management of patients. The purpose of this paper is to provide an overview of the main imaging features of adrenal masses and tumor-like conditions recalling the strengths and weaknesses of imaging modalities commonly used in adrenal imaging.

## Keypoints

- Adenoma is the most common adrenal tumor
- Washout evaluation of adrenal masses on CECT is the gold standard technique
- Chemical shift MR imaging is a useful tool to identify intra-lesional fat content
- DWI is not useful to differentiate adrenal lesions
- Nuclear medicine techniques are helpful in some specific settings

## Introduction

The adrenal gland is a site of several pathologic conditions, including hyperplasia, hemorrhage, and malignant and benign masses. Tumors can be hyperfunctioning, when producing an increased amount of hormones leading to endocrine disorders, or non-hyperfunctioning, which are characterized by normal hormone levels. Therefore, in some cases, these lesions can be clinically suspected, but in most cases adrenal masses are discovered incidentally. Indeed, the increasing use of imaging investigations, including ultrasound (US), computed tomography (CT), and magnetic resonance (MR), has led to an increase in the detection of such incidental lesions [1–4]. Incidental adrenal masses are common, occurring in about 3–7% of adults [3], with the majority of them being benign non-functioning adenomas [4]. Characterization of an adrenal mass as benign or malignant is critical and imaging plays a key role in influencing the clinical management of patients. In this setting, the role of radiologists is crucial in both the choice of imaging modality and protocol technique to be used and interpretation of imaging findings. US has a limited part in the assessment of adrenals due to its low accuracy in the detection and characterization of small adrenal neoplasms, in comparison to CT and MR, being useful to differentiate a cystic from a solid mass. Nevertheless, US has still a major role in neonatal patients in which the adrenals are bigger compared to the kidneys. CT represents the first-level imaging modality for the evaluation of adrenal lesions, since it permits a quick execution ensuring high spatial resolution, with findings of pre-contrast images and post-contrast behavior being commonly used to achieve a correct diagnosis [5]. MR still remains a second-level technique, although its advantages include multiparametricity, multiplanarity, higher contrast resolution in comparison with CT, and the added value of chemical shift imaging (CSI) [6].

Both CT and MR have therefore a pivotal role in adrenal imaging. When the clinical laboratory picture shows a suspicious functioning adrenal lesion, nuclear medicine techniques such as scintigraphy with ^131/123^I-meta-iodo-benzyl-guanidine (MIBG) and Octreotide can be used. Moreover, positron emission tomography (PET) has also shown to be helpful for the evaluation of non-hyperfunctioning adrenal masses with high accuracy in the characterization of malignant lesions [7].

The purpose of this paper is to give an overview of the imaging features of adrenal masses and tumor-like conditions with an emphasis on CT and MR. We will also briefly discuss the recent advancements in diagnostic radiology and nuclear medicine modalities.

### Anatomy

The adrenals are paired retroperitoneal glands that lie superiorly and anteromedially to the kidneys. They are triangular-shaped with the right adrenal gland being usually more pyramidal in shape [8]. The adrenal glands are made of an outer cortex deriving from the mesoderm and an inner medulla deriving from the neural crest. In the cortex, the zona glomerulosa produces mineralocorticoids, the zona fasciculata glucocorticoids, and the zona reticularis sex steroids or gonadocorticoids, whereas the medulla produces catecholamines, adrenaline, and noradrenaline. The adrenals are supplied by three arteries: superior (from the inferior phrenic arteries), middle (from the abdominal aorta), and inferior suprarenal arteries (from the renal arteries). The right adrenal vein ends into the inferior vena cava, while the left adrenal vein ends into the left renal vein or inferior phrenic vein. The lymphatic vessels drain to the para-aortic nodes. The innervation of adrenals comes from the splanchnic nerves arising from the aortic and renal plexuses [9].

### Benign adrenal tumors and tumor-like conditions

#### Cortical adenoma

Adenoma is the most common benign adrenal tumor, with no malignant evolution, arising from the cortex and consisting of clear cells with abundant intracytoplasmatic fat, which is the key element for the differential diagnosis with malignant neoplasms at imaging. Typically, the remaining portion of the adrenal gland and the contralateral adrenal appear atrophic. Large adenomas may show cystic components, calcifications, and hemorrhagic areas [10].

Incidence of adenomas increases with age and most of them are non-functioning, with these lesions being generally asymptomatic and incidentally discovered [11]. Hyperfunctioning adrenal adenomas present with symptoms and signs related to excess hormone secretion, thereby determining Cushing’s syndrome (hyperproduction of cortisol) or Conn’s syndrome (hyperproduction of aldosterone), while they rarely lead to an adrenal-genital syndrome [12].

The typical adenoma measures less than 3 cm making challenging its detection through US, in which it appears as a homogeneous and hypoechoic solid lesion with well-defined margins, hypovascularization on Color Doppler examination [13], and hypoenhancing on contrast-enhanced US (CEUS) [14]. At unenhanced CT, adenomas are usually well-demarcated round or oval lesions, with homogeneous and relatively low attenuation values (lower than 10 Hounsfield Units [HU]), due to high fat content [15]. However, unenhanced CT alone is not always diagnostic since 15–30% of adenomas are lipid-poor, thereby showing higher attenuation values. In these cases, additional imaging after intravenous contrast administration is required to differentiate adenoma from non-adenomas. The evaluation of the enhancement pattern of adrenal lesions needs a further late phase after the acquisition of the venous phase. There are different CT protocols suggested for the evaluation of adrenal masses; however, there is evidence that a 15-min post-contrast protocol has the highest diagnostic accuracy [16–20].

Absolute percentage washout (APW) is calculated using the following formula:

$$ \left(\mathrm{enhanced}\ \mathrm{HU}-15-\min\ \mathrm{delayed}\ \mathrm{HU}\right)/\left(\mathrm{enhanced}\ \mathrm{HU}-\mathrm{unenhanced}\ \mathrm{HU}\right)\times 100\% $$

Relative percentage washout (RPW) is used when unenhanced CT value is not available, and the enhanced values are compared with 15-min delayed scans, by using the following formula:

$$ \left(\mathrm{enhanced}\ \mathrm{HU}-15-\min\ \mathrm{delayed}\ \mathrm{HU}\right)/\left(\mathrm{enhanced}\ \mathrm{HU}\right)\times 100\% $$

If the APW is > 60% or RPW is > 40% after 15-min from contrast administration, this is indicative of adenoma, with sensitivity and specificity of 88% and 96% at the APW and sensitivity and specificity of 83% and 93% at the RPW, respectively [5]. This method enables to differentiate adenomas, which enhance quickly and show rapid washout, from non-adenomas such as metastases, which instead demonstrate strong enhancement but prolonged washout. Another useful imaging feature is the size, since lesions greater than 4 cm lay for malignant tumors, and the dimensional growth, considering that adenomas grow slower than malignant masses [21].

**Fig. 1:**
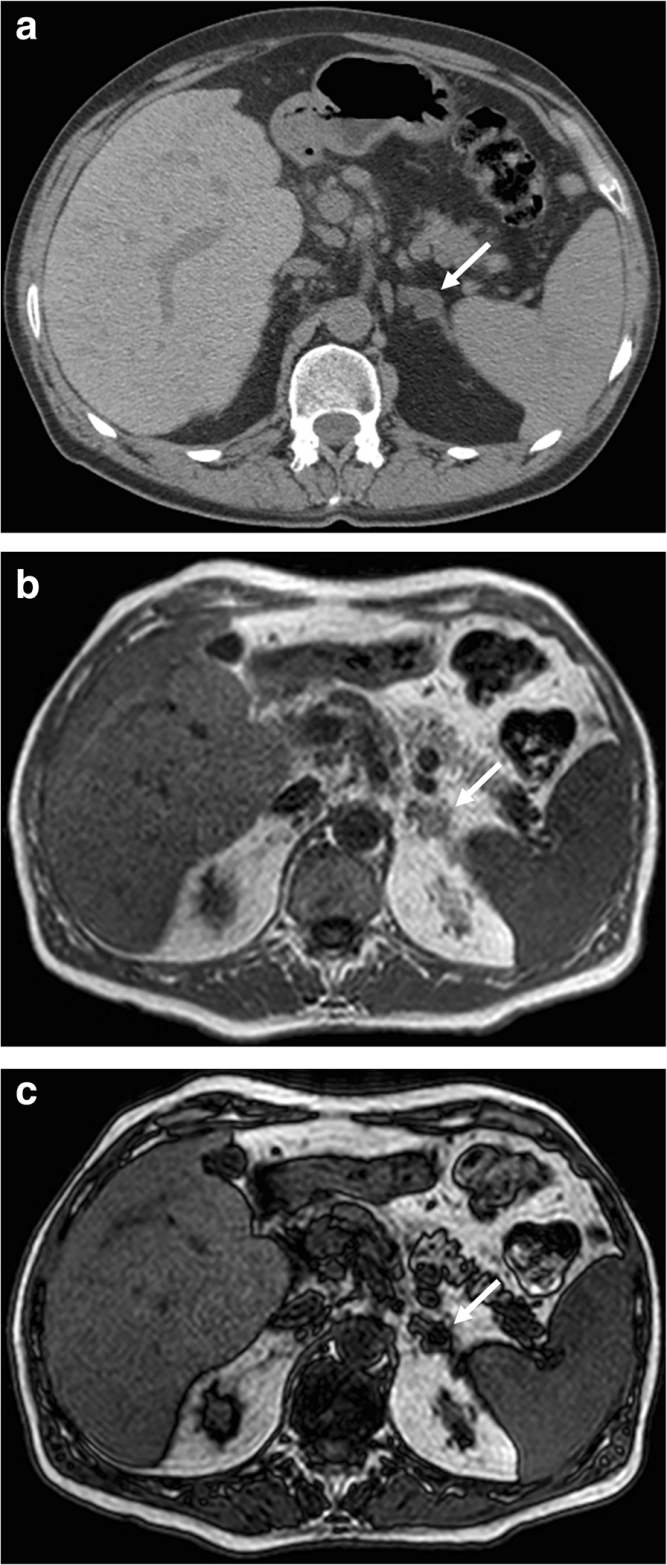
Adrenal adenoma of a 45-year-old woman. Unenhanced CT (**a**) shows a left adrenal mass (arrow) with lower attenuation values than 10 HU. Axial T1-weighted in-phase (**b**) and out-of-phase (**c**) gradient recalled echo MR images show prominent loss of signal on out-of-phase

MR has similar diagnostic accuracy to CT allowing characterizing adenomas regardless of their CT enhancement (Fig. 1) [6]. On T2-weighted MR images, adrenal adenomas are homogeneous and present intermediate-low signal intensity compared to skeletal muscle or liver; intra-lesional hemorrhage can occur in large adenomas resulting in hyperintense areas on T1-weighted images [20]. An important component of adrenal MR protocol is CSI. This modality uses the different precession frequencies of protons in both water and fat within the same voxel and creates in-phase and opposed-phase images, which enable to detect intra-lesional fat resulting in a loss of signal intensity in the opposed phased images [6]. Thus, most adrenal adenomas demonstrate a loss of signal intensity on out-of-phase images, and a decrease in signal intensity of more than 20% is considered diagnostic of adenoma. The MR sensitivity for adenomas measuring 10–20 HU is nearly 100%, while that for lipid-poor adenomas measuring greater than 30 HU is significantly lower (13–75%) [10, 22]. The loss of signal intensity can be also quantified using parameters such as the spleno-adrenal ratio (ASR) and the signal intensity index (SII). An ASR < 70% is highly specific for adenoma and has sensitivity of 78%; while using SII, a loss of signal intensity greater than 5% has allowed to identify adenomas in 100% of cases [23]. Of note, Namimoto et al. reported similar results in the identification of adenomas by comparing three-point Dixon techniques for CSI and conventional dual-echo imaging [24].

In comparison to contrast-enhanced CT (CECT), contrast-enhanced MRI with gadolinium washout studies does not appear to exhibit the same diagnostic strength. Thus, CECT remains the gold standard technique, especially in the evaluation of lipid-poor adenomas. Data related to diffusion-weighted imaging (DWI) of adrenal masses have been disappointing mainly due to benign adrenal adenomas’ propensity to demonstrate restricted pattern of diffusion. Miller et al. retrospectively evaluated 160 adrenal lesions and found that apparent diffusion coefficient (ADC) was not useful to differentiate benign from malignant adrenal neoplasms [25]. Sandrasegaran et al. also found that ADC values were not helpful for this purpose [26].

Over the last years, CT perfusion imaging has been widely used for the diagnosis and characterization of tumors, but few studies about its potential role in the characterization of adrenal lesions have been reported. Perfusion imaging evaluates tissue vascularity after contrast media administration by measuring changes in tissue enhancement and perfusion. Previous studies have showed that a perfusion parameter indicated as blood volume is significantly higher in adenomas, being potentially helpful to differentiate adenomas from non-adenomas [27].

Regarding the role of dual energy CT, studies have reported that this imaging modality allows an accurate characterization of lipid-rich adenoma evaluating that a decrease in attenuation of an adrenal lesion between 140 and 80 kVp is a highly specific sign of adenoma. However, because an increase in attenuation at 80 kVp is seen on metastatic lesions and some adenomas, the sensitivity of this modality is still low [28].

Few data have been published on nodule characterization by histogram analysis, thus there is poor knowledge regarding the potential application of this tool. This method also takes advantage of the higher intracytoplasmatic fat content of adenomas. With a threshold value of > 10% negative pixels, it is possible to identify many benign adrenal nodules with attenuation values > 10 HU on unenhanced CT with high accuracy; moreover, a threshold of > 10% negative pixels seems to guarantee 100% specificity [29]. New interesting perspectives include the characterization of adrenal lesions by using texture analysis, with some reports already showing promising results by some texture analysis-derived features [30].

#### Hyperplasia

Hyperplasia refers to nonmalignant diffuse or focal enlargement of the adrenal gland, which is an uncommon cause of ACTH-independent Cushing’s syndrome or Conn’s syndrome. Adrenal hyperplasia can be congenital or acquired and is classified according to morphology in diffuse or nodular adrenal hyperplasia [31]. Among the causes of ACTH-independent Cushing’s syndrome, we should recall primary pigmented nodular adrenal disease (PPNAD), which is a benign condition generally characterized by mild symptoms related to hypercortisolism. In patients with PPNAD, imaging allows to identify multiple small nodules in normal-sized or enlarged adrenal glands [32].

**Fig. 2:**
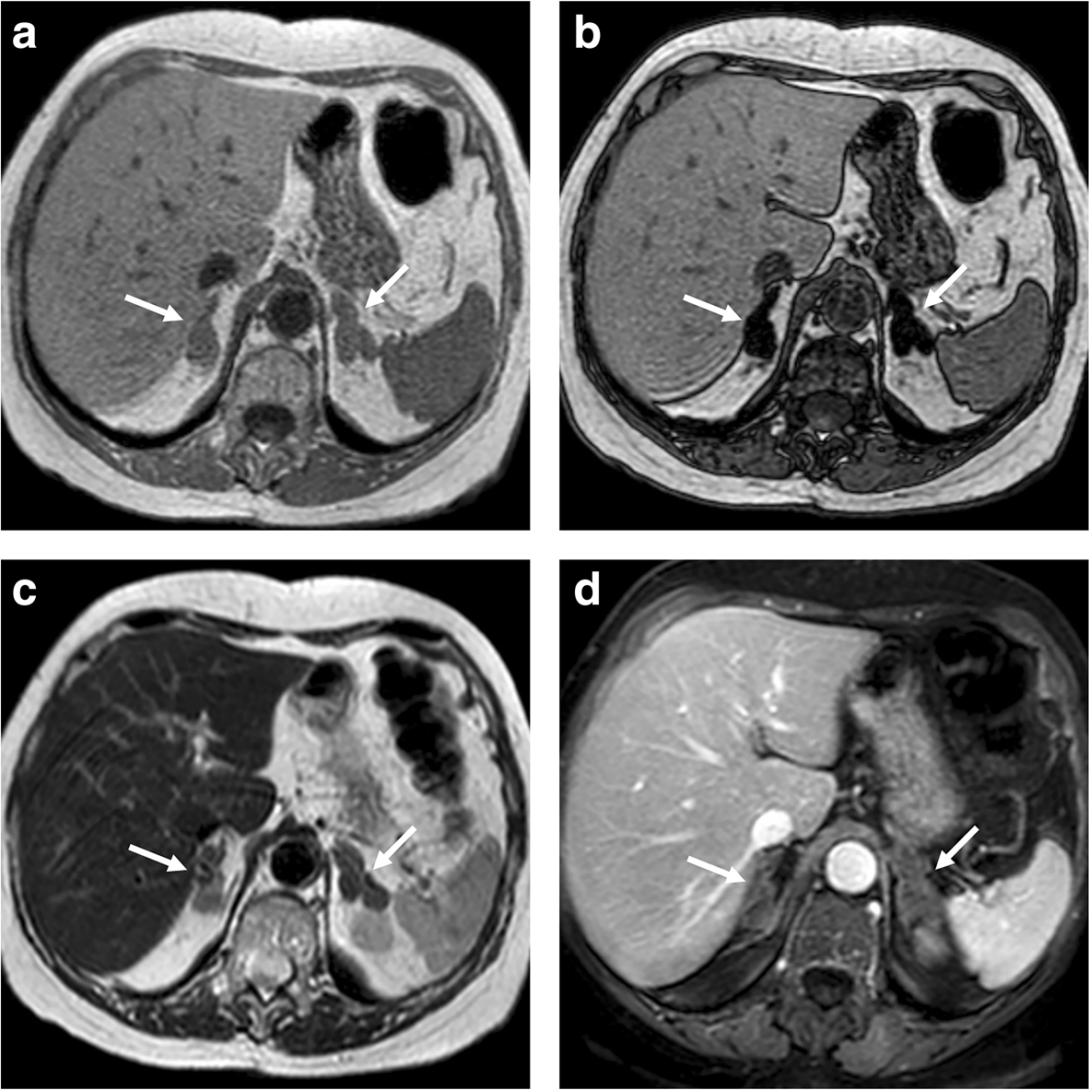
Bilateral adrenal hyperplasia in a 75-year-old woman. Axial T1-weighted in-phase (**a**) and out-of-phase gradient recalled echo (**c**), T2-weighted fast spin echo (**c**), and three dimensional fat-suppressed T1-weighted gradient recalled echo image in portal phase after contrast injection (**d**) show the nodular enlargement of both adrenal glands (arrows) with loss of signal on out-of-phase (**b**)

At CT, diffuse hyperplasia is characterized by the increased volume of both adrenals presenting smooth margins, a homogeneous structure, and often some nodules of variable size, usually smaller than 5–10 mm, but even up to 2 cm. A large nodule is rarely detected and it differs from the adenoma because the former lies within enlarged glands, while the latter appears within an atrophic adrenal gland or associated with atrophy of the contralateral gland [33]. Hyperplasia contains variably sized groups of lipid-rich cells, thereby showing a significant overlap with adenoma in terms of lesion attenuation and APW or RPW [33]. On MR, hyperplasia shows signal intensity similar to that of the normal gland but appears as homogeneous or nodular enlarged gland; this appearance helps in the differentiation from other nodular pathologies, which are generally hyperintense on T2-weighted images (Fig. 2) [34].

#### Adrenal infection

Tuberculosis is one the most frequent adrenal infections, still being the primary cause of primary adrenal insufficiency in developing countries. Generally, tuberculosis affects both adrenal glands and the clinical picture is that of adrenal insufficiency, including weakness, nausea, anorexia, weight loss, abdominal pain, and hypotension. Depending on the disease stage, CT and MR show different findings. At first, a mass-like enlargement with preserved contour is commonly detected. Central low attenuation on unenhanced CT and peripheral enhancement on post-contrast CT and MR images is another typical imaging finding due to a central caseous necrosis area [35]. Later, a decrease in size of adrenal glands is observed with the frequent occurrence of diffuse, localized, or punctuated calcifications, with atrophic adrenal glands with calcifications being considered highly suspicious for chronic tuberculosis [36]. During antituberculosis therapy, the caseous necrosis can be replaced by fibrous tissue and cicatrix, thereby leading to a more homogeneous enhancement or central calcific deposits [36]. Another adrenal infection mimicking tuberculosis is histoplasmosis, although neoplastic conditions such as metastases or lymphoma may show similar imaging features. Thus, histologic evaluation of adrenal tissue after biopsy is generally required to achieve the correct diagnosis.

#### Adrenal hemorrhage

Adrenal hemorrhage is a condition with different etiopathogenetic, clinical, and prognostic aspects depending on whether it occurs in children or in adults. Adrenal hemorrhage can result from trauma, anticoagulant therapy, complicated pregnancy, sepsis, or stress, such as surgery [37, 38]. Moreover, adrenal hemorrhage may occur within adrenal lesions such as adenoma, myelolipoma, pheochromocytoma, metastasis, and cortical carcinoma. Trauma is the most common cause, with post-traumatic hemorrhage being usually unilateral [37]. Bilateral hemorrhage occurs in 20% of cases, and adrenal insufficiency secondary to hemorrhage is extremely rare [38]. US represents the first-level imaging modality in children. Acute hemorrhage appears as a mass with a diameter of 3–4 cm with a mixed echostructure, predominantly hyperechogenic due to blood clots within the hematoma. In the subsequent phases, the mass progressively becomes liquid and then hypo-anechoic due to the lysis of the clots. At Color Doppler, adrenal hemorrhage shows no vascularization [38].

**Fig. 3:**
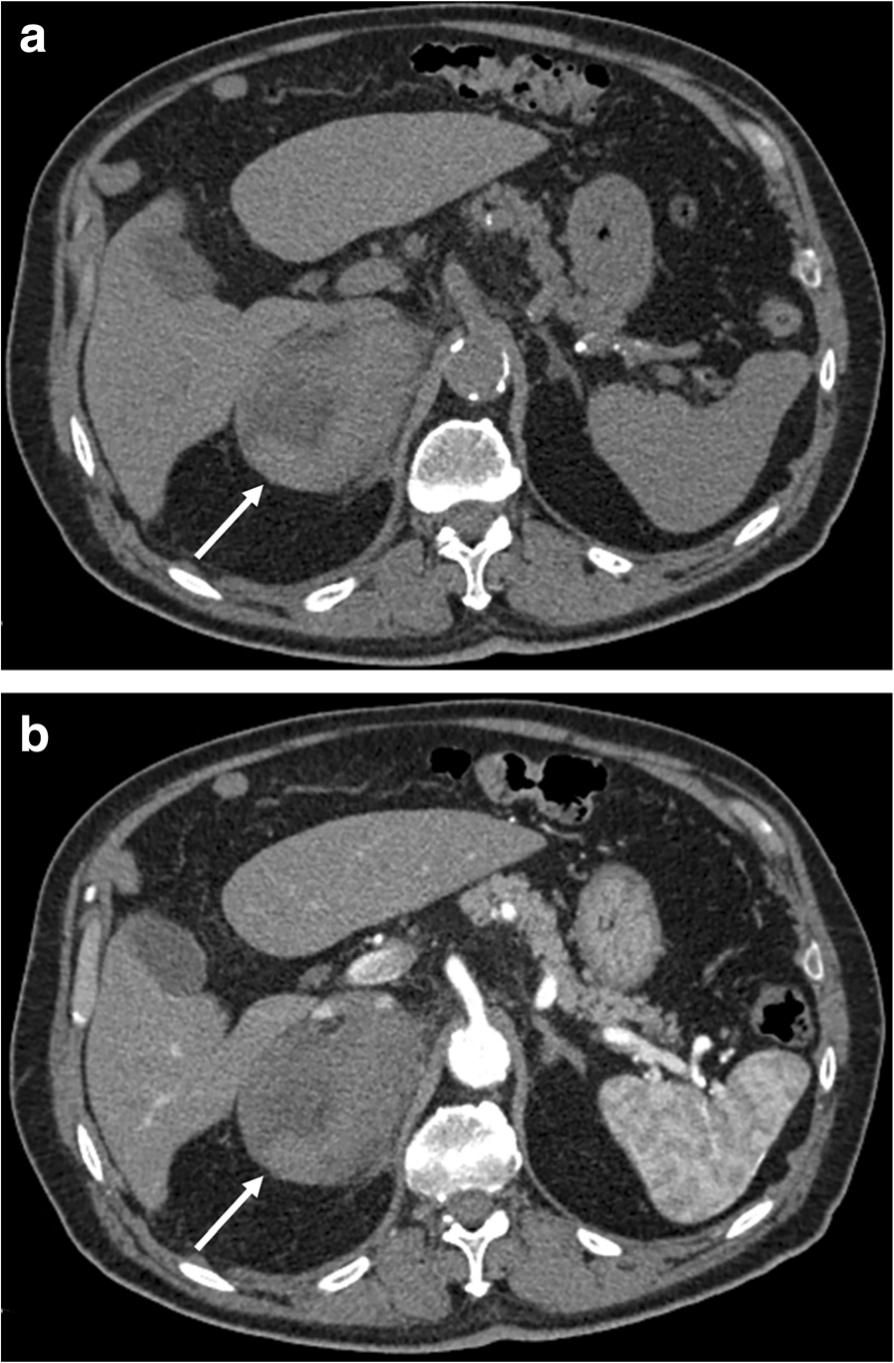
Right adrenal hemorrhage in a 61-year-old man with acute abdominal pain after starting anticoagulant therapy. Axial unenhanced (**a**) and arterial (**b**) phase CT images show a large right adrenal hematoma (arrow) with relatively high attenuation values and without enhancement after contrast injection

On CT, acute hemorrhage is characterized by high attenuation values, in post-traumatic cases, associated with hemorrhagic suffusion of periadrenal fat and retroperitoneal bleeding (Fig. 3). A chronic hematoma appears as a mass with a hypoattenuating center with or without calcifications, also termed adrenal pseudocyst. Hematomas usually decrease in size and may spontaneously disappear. Calcifications may develop in the late stage of hemorrhages [38].

On MR, signal intensity of adrenal hemorrhage depends on its stage. In the acute stage (first 7 days), the hematoma appears isointense or slightly hypointense on T1-weighted and hypointense on T2-weighted images, due to the presence of deoxyhemoglobin. In the subacute phase (1 week to 7 weeks), the hemorrhage appears hyperintense on both T1- and T2-weighted images due to the paramagnetic effect of the methemoglobin. In the chronic stage (more than 7 weeks), a hypointense rim on T1- and T2-weighted images due to hemosiderin deposition and fibrosis is detectable [38].

#### Cyst

**Fig. 4:**
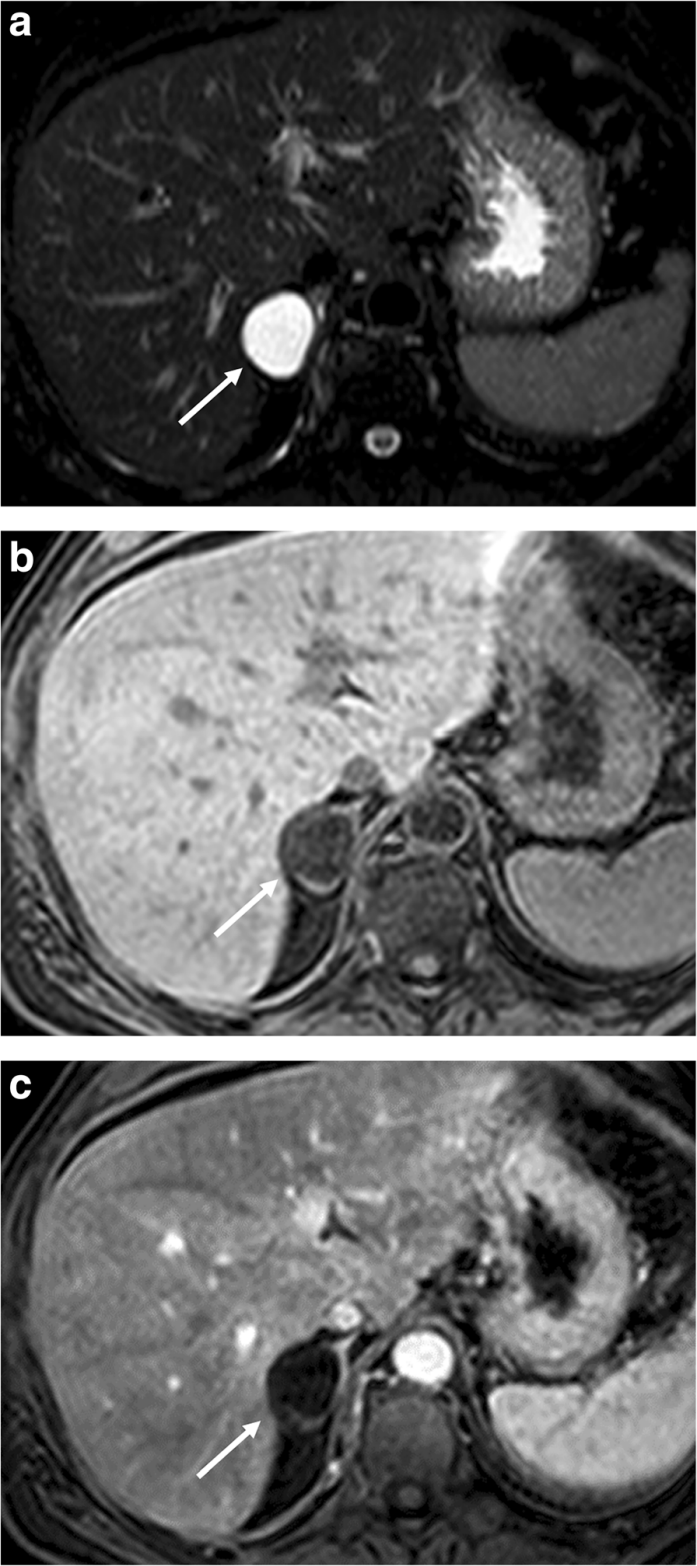
Right adrenal cyst in a 50-year-old woman. Axial T2-weighted fat-saturated fast spin echo MR image (**a**), three-dimensional fat-suppressed T1-weighted gradient recalled echo images before (**b**) and after paramagnetic contrast administration (**c**) show a right adrenal cystic lesion (arrow) with low T1 signal intensity, high T2 signal intensity, no contrast enhancement and no septations, blood products, soft-tissue components, or calcifications

Adrenal cysts are uncommon. Traditionally, adrenal cysts have been classified as pseudocysts (resulting from previous hemorrhage), endothelial cysts (with a thin endothelial wall and lactescent liquid, deriving from lymphangiectasia or arteriovenous malformations), epithelial cysts (with thin epithelial wall and serous liquid content), and parasitic cysts (hydatid disease). These cysts can range in size from 1 to 20 cm [39, 40]. At US, endothelial and epithelial cysts appear as well-circumscribed hypoechoic or anechoic lesions with thin walls, while pseudocysts may have thicker walls. The scattered echogenicity, the presence of debris, or fluid-fluid levels suggest previous hemorrhage into a pseudocyst [40]. On CT, adrenal cysts appear as well-demarcated, non-enhancing, hypoattenuating lesion with water attenuation (< 20 HU) and thin walls. Nevertheless, pseudocysts may have a minimally thick cyst wall and complicated fluid [31]. In some cases, internal hemorrhage or calcifications can be detected, especially in pseudocysts and parasitic cysts. On MR, cysts are usually hyperintense on T2-weighted images (Fig. 4). Septations, blood products, or a soft-tissue component and calcifications may be observed in pseudocysts [31].

#### Myelolipoma

Adrenal lipomatous tumors are neoplasms with a significant proportion of adipose tissue, with myelolipoma being the most common type. Myelolipoma is a benign tumor, usually unilateral, small, and often incidentally discovered. It consists of mature fat and hematopoietic cells from myeloid, erythroid, and megakaryocytic lines. The amount of fatty component is variable with some tumors showing small regions of fat within a predominantly soft-tissue mass, whereas others consist almost completely of fat. It can infrequently contain calcifications or show partial replacement by hemorrhage or fibrosis. Large lesions tend to become symptomatic due to pressure effects and to intra-lesional hemorrhage or infarctions. While smaller lesions can be managed conservatively, those with a size of more than 4 cm are likely to be surgically removed. Indeed, the American Association of Clinical Endocrinologists and American Association of Endocrine Surgery (AACE/AAES) 2009 guidelines recommend adrenalectomy for all myelolipomas measuring more than 4 cm [41].

At US, fat-predominant lesions appear hyperechoic, while myelolipomas mainly composed of myeloid elements appear hypoechoic. Frequently, the lesion has mixed hyperechoic and hypoechoic areas, due to varying amounts of fat and myeloid elements [42].

At CT, myelolipoma appears as a round mass with well-defined borders. The density varies depending on the proportion of fat and myeloid components. Internal inhomogeneity is related to intra-lesional septa, necrosis, hemorrhage, or calcifications. Thus, myelolipoma goes from totally fat lesions with thin circles of parenchymatous tissue to clearly parenchymatous forms with small areas of fat. At CT, myelolipoma is easily diagnosed because gross fat, with attenuation values < − 30 HU, is almost always detectable. After contrast agent injection, myelolipoma typically shows slight enhancement because it is poorly vascularized [31, 42].

**Fig. 5:**
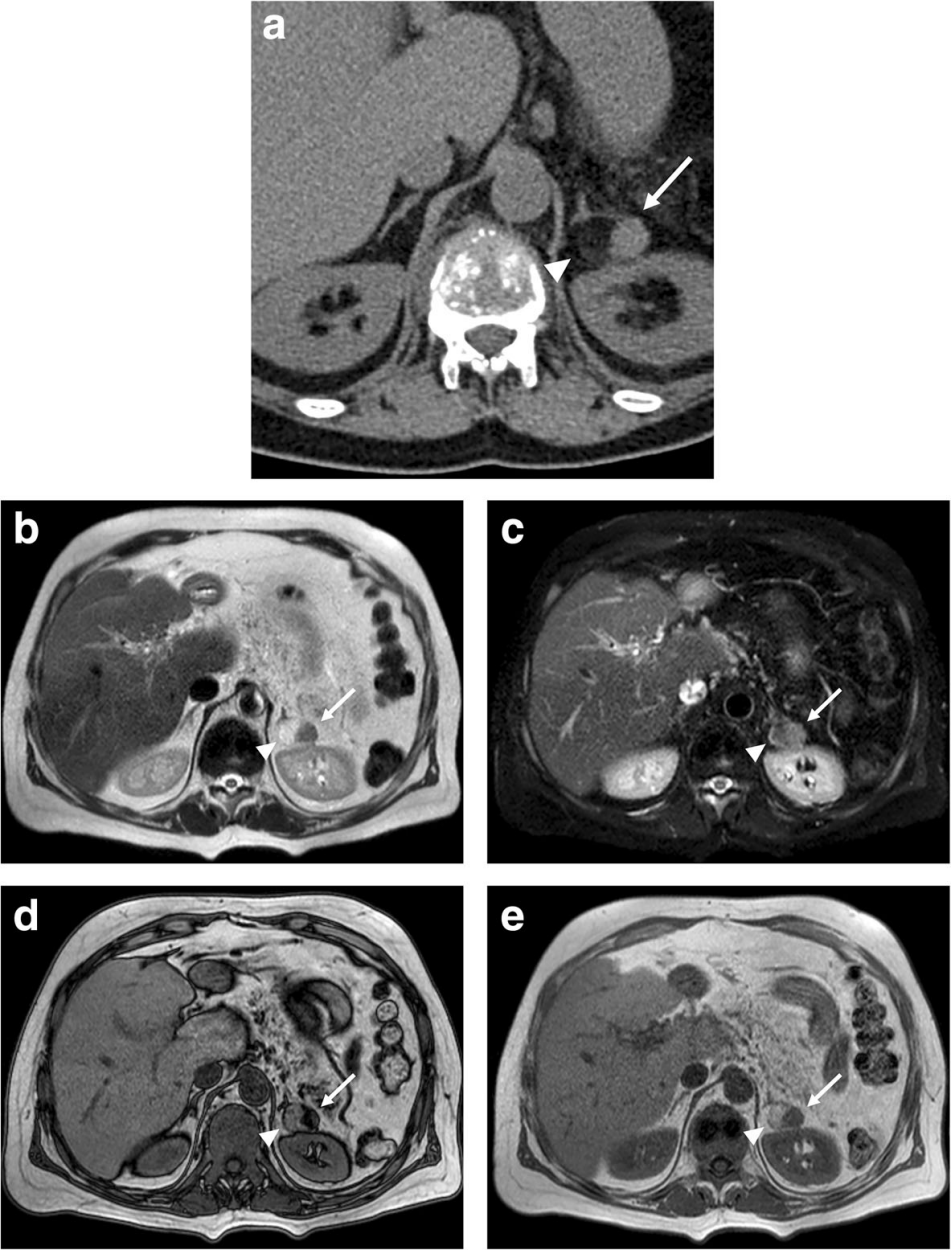
Left adrenal myelolipoma incidentally discovered in a lumbar CT scan on a 51-year-old man. Unenhanced CT (**a**) shows a left adrenal mass with macroscopic fat (arrowhead) and a soft-tissue myeloid component (arrow). Axial T2-weighted fast spin echo (**b**), T2-weighted fat-saturated spin echo (**c**), T1-weighted in-phase (**d**), and out-of-phase gradient recalled echo images (**e**) show the signal drop of macroscopic fat in fat-suppressed T2-weigthed images (**c**, arrowhead) and show loss of signal on out-of-phase imaging of the myeloid element (**e**, arrow)

On MR, the fat macroscopic components are hyperintense on T1- and T2-weighted images, while the hematopoietic parts are hypointense on T1- and moderately hyperintense on T2-weighted images. The diagnosis becomes certain when the hyperintensity on T1-weighted images disappears on sequences with fat suppression [41, 42]. Similar to renal angiomyolipomas, the presence of the India ink artifact on CSI at the myelolipoma-adrenal interface or within an adrenal mass on opposed-phase images should indicate a myelolipoma [43]. Fat-suppressed images show greater loss of signal intensity than CSI sequences, as the presence of macroscopic adipose tissue is typical of myelolipoma (Fig. 5). On contrast-enhanced MR, myelolipoma may have enhancement of the myeloid component. Based on MR characteristics, three different types of myelolipoma were described. The first is mostly composed of fat and homogeneous and hyperintense on T1-weighted images. The second consisted of similar content of fat and myeloid components with a heterogeneous appearance. The third type is mainly composed of myeloid tissue with enhancement after contrast injection [9].

#### Lipoma

Adrenal lipomas are rare tumors of mesenchymal origin containing mature fatty tissue and surrounded by a fibrous capsule, similarly to lipomas elsewhere in the body, with only a few cases having been reported in the adrenals [44, 45]. These are well-demarcated lesions composed of lobules of fat tissue. On CT, in contrast to myelolipomas, they show no or minimal soft-tissue attenuation and can contain focal areas of intra-lesional calcifications due to degenerative changes [44, 45].

#### Pheochromocytoma

Pheochromocytoma arises from the chromaffin cells of the adrenal medulla and typically produces both norepinephrine and epinephrine [46]. Extra-adrenal pheochromocytomas are uncommon and are termed paragangliomas, originating in the sympathetic ganglia at the level of the mediastinum, abdomen, and pelvis. Adrenal pheochromocytoma is commonly benign, although 10% of these lesions can be malignant [46]. Malignant pheochromocytomas are recognized for local infiltration or metastases, usually involving the bone, liver, lymph nodes, lungs, and brain. Clinical symptoms are associated with catecholamine excess (headache, sweating, palpitations, pallor, weight loss, and/or anxiety). This tumor is generally sporadic, although it can be associated with hereditary syndromes including multiple endocrine neoplasia (MEN)-2A, MEN-2B, von Hippel-Lindau, and NF-1. Pheochromocytomas are variable in size ranging from 1.2 to 15 cm with a mean size of 5.5 cm [13, 31]. When a pheochromocytoma is suspected, the first diagnostic step is the evaluation of catecholamines and metanephrines in urine.

The diagnosis of pheochromocytoma at imaging is often challenging due to its complex and variable appearance, related to necrosis, fibrosis, cystic and fatty degeneration, and calcification, which has led to describe it as “imaging chameleons” mimicking other lesions [47]. At US, pheochromocytomas are heterogeneous and well-encapsulated, with hypervascularization at color Doppler and an early arterial pattern of enhancement at CEUS [48, 49].

At CT, pheochromocytomas may present solid, cystic, calcific, and/or necrotic components. Smaller tumors tend to display a more uniform attenuation, with a density of 40–50 UH [47, 49]. After contrast administration, pheochromocytomas enhance avidly with some of them showing higher enhancement on the portal venous phase and other on the arterial phase; nevertheless, their APW and RPW are similar to those of adenomas [49]. Therefore, pheochromocytomas often cannot be reliably differentiated from adenomas using CT washout protocols. When lesions are quite large (> 6 cm), intra-lesional hemorrhage, necrosis, or calcifications can be observed, determining the inhomogenous contrast enhancement.

**Fig. 6:**
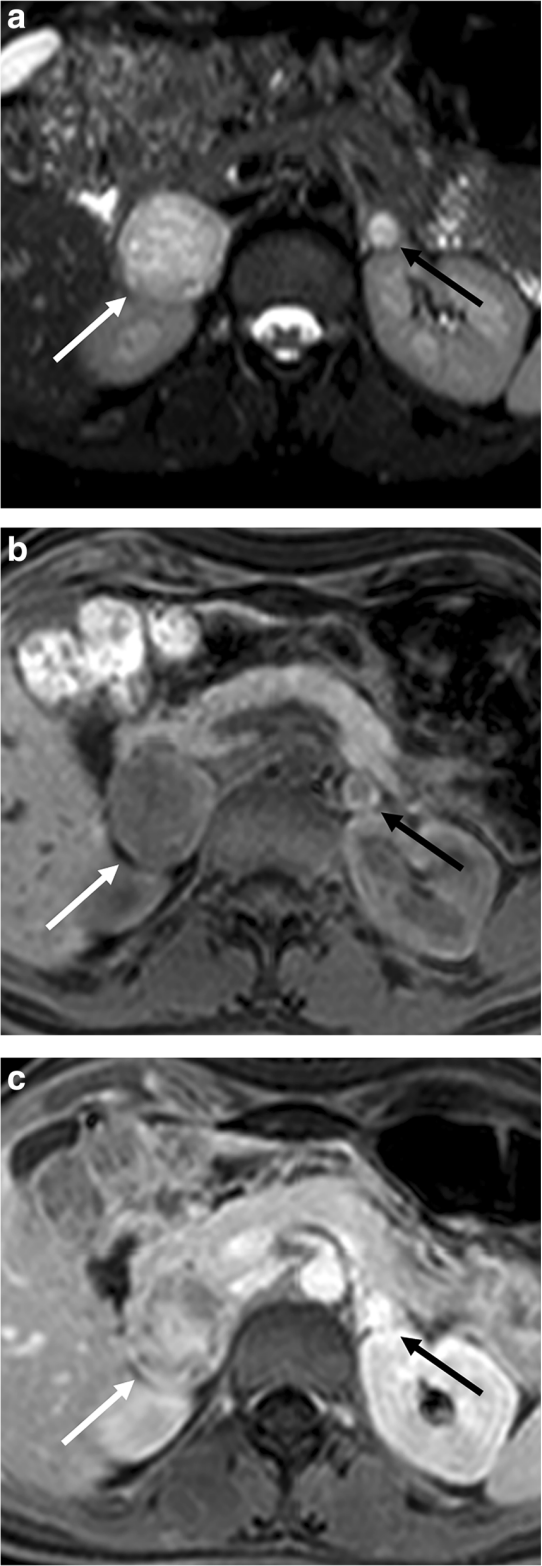
Bilateral adrenal pheochromocytoma in a 27-year-old woman with multiple endocrine neoplasia type 2. Axial T2-weighted fat-saturated spin echo (**a**), three-dimensional fat-suppressed T1-weighted gradient recalled echo images before contrast injection (**b**), and in portal phase (**c**) images show a bilateral adrenal mass with high T2 signal intensity and strong and heterogenous contrast enhancement. The signal intensity is typically more inhomogenous in larger lesions (white arrow, right adrenal gland) than in smaller ones (black arrow, left adrenal gland)

MR is highly sensitive for the diagnosis of pheochromocytomas with a sensitivity of about 98% [49]. Pheochromocytoma is slightly hypointense on T1-weighted and hyperintense on T2-weighted images [50]. It does not present loss of signal intensity on opposed-phase images, unlike the typical adenoma (Fig. 6). In some cases, areas of fatty degeneration can be encountered, leading to slight signal drop on CSI [6]. Although DWI has not a key role in the differentiation of benign and malignant adrenal lesions [25, 26], it is potentially useful to detect pathologic lymph nodes and liver metastases. Nevertheless, a study performed at 3 T has reported significantly lower ADC values of malignant pheochromocytomas in comparison to benign ones [51]. MR spectroscopy has also been used to characterize adrenal masses and particularly pheochromocytomas, postulating that metabolic content can be measured using that modality. Specifically, it was found that pheochromocytomas exhibit a unique spectral appearance, with the most prominent resonating peak at 6.8 ppm, with other resonance peaks at 2.7, 3.16, and 3.8 ppm, attributable to catecholamines and catecholamine-derived metabolites. However, the use of this technique has been remarkably limited by the anatomical location of the adrenal glands, as their vicinity to the diaphragm makes the analysis quite demanding [52]. ^131/123^I-MIBG scintigraphy is currently the functional nuclear medicine imaging of choice for pheochromocytoma [11] but suffers from drawbacks like limited spatial resolution, difficulty in detection of small tumors (< 1.5–2.0 cm) or large tumors with extensive necrosis/hemorrhage, lack of tracer uptake in some tumors, and interference with certain medications, leading to false-negative results [53]. Expression of somatostatin receptors by pheochromocytoma facilitates targeted PET imaging with ^68^Ga-DOTA-peptides. In a study of Sharma et al., ^68^Ga-DOTANOC PET/CT showed high diagnostic accuracy (on patient-based analysis sensitivity, specificity, and accuracy of 92%, 85%, and 90%, respectively) in patients with a suspicion of pheochromocytoma, and was superior to MIBG imaging [54]. Furthermore, the properties of uptake and storage of catecholamines in pheochromocytomas open the potential use of ^18^F-FDOPA PET/CT for the evaluation of the relationship between tumor secretion and its biochemical phenotype [55].

#### Hemangioma

**Fig. 7:**
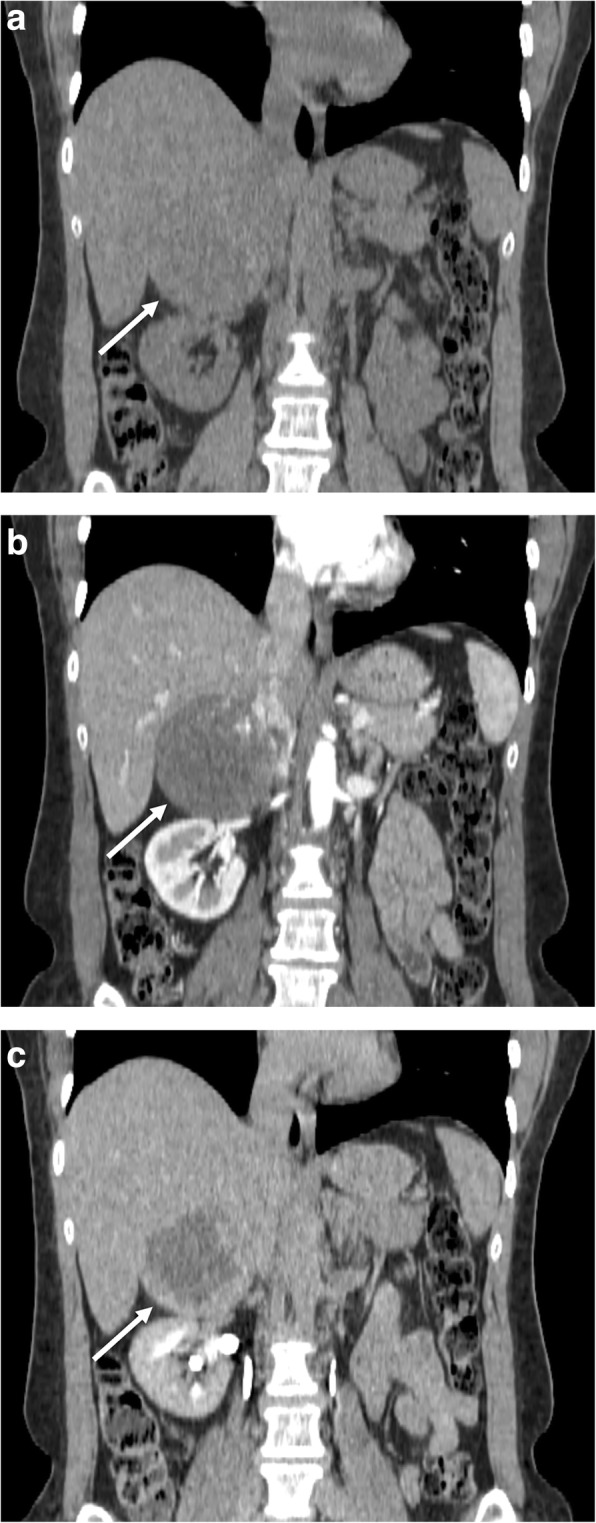
Right adrenal hemangioma in a 49-year-old woman. Coronal reformatted CT images before (**a**) and after contrast injection in arterial (**b**) and late phase (**c**) show a well-encapsulated large right adrenal lesion (arrow) showing peripheral contrast enhancement with progressive centripetal filling and a hypodense center

Hemangioma is an uncommon incidental adrenal lesion generally discovered by CT or MR, especially in women between 40 and 70 years of age. Regarding the histologic types, the most common are capillary and cavernous hemangiomas [56]. Its radiologic features are similar to those of hepatic and soft-tissue hemangiomas. On US, hemangioma has no specific characteristics, appearing as a mass with variable size and echotexture. On CT, small hemangiomas can be homogeneous, whereas larger lesions present calcifications which can be either phleboliths or dystrophic calcifications due to previous hemorrhages [31]. On CECT, hemangioma shows heterogeneous and mainly peripheral enhancement, with or without central filling on delayed phases (Fig. 7) [31]. At MR, hemangioma is hypointense on T1-weighted with a remarkable hyperintensity on T2-weighted images (lightbulb sign). Hypointense foci can be encountered on T1- and T2-weighted images related to intra-lesional calcifications. Also on contrast-enhanced MR, hemangioma usually displays peripheral nodular enhancement.

#### Lymphangioma

Lymphangiomas are rare adrenal incidentally discovered benign lesions, being totally asymptomatic and occurring at any age [57]. On CT, lymphangioma appears as a well-encapsulated hypodense lesion (8–20 HU) with an average size of 3 cm with intra-lesional septations showing contrast enhancement. Lymphangioma can also contain calcifications which appear scattered punctate or thick curvilinear [58]. At MR, lymphangioma does not display specific imaging features showing low signal intensity on T1- and high signal on T2-weighted images.

#### Schwannoma

Schwannoma is a rare and usually benign tumor arising from the sheath of the nerves, with only few cases of adrenal schwannomas having been previously described [59]. This lesion is commonly asymptomatic, but in some cases it may present with abdominal pain, generally due to hemorrhage. At CT, it appears as hypodense with inhomogeneous contrast enhancement. Larger lesions usually present degenerative changes including hemorrhagic components, calcifications, and cystic areas, making MR features non-specific, although adrenal schwannoma generally appears isointense on T1-weighted images showing slight hyperintensity on T2-weighted images [59, 60].

#### Ganglioneuroma

Ganglioneuroma is a benign tumor arising from the sympathetic nerves or within the adrenals. This lesion is more frequently detected in the mediastinum or retroperitoneum than in the adrenal medulla [61]. Usually, it is a small round mass (2–3 cm) with well-defined smooth margins and an inhomogeneous appearance due to mixoid components. On CT, it appears as a well-circumscribed solid iso- or hypoattenuating lesion, which may display calcifications, necrosis, and hemorrhagic areas [62]. It remains hypoattenuating on early post-contrastographic phases, while becoming hyperattenuating on delayed phases due to persistent enhancement [62]. On MR, ganglioneuroma is hypointense on T1-weighted and hyperintense on T2-weighted images, with slow and persistent enhancement on post-contrastographic images [62].

#### Adenomatoid tumor

Adenomatoid tumor is an uncommon benign lesion with mesothelial origin, which is generally asymptomatic. On CECT, this tumor appears as a hypoattenuating lesion with heterogeneous contrast enhancement. Calcifications and septations may be observed within the mass. MR findings are non-specific with a variable appearance on T1-weighted, T2-weighted, and post-contrast images [31].

#### Oncocytoma

Oncocytoma is a rare epithelial tumor affecting more frequently the left adrenal and showing a mean size of about 9 cm. This neoplasm is generally benign and non-functioning, although few cases of functioning lesions secreting cortisol and androgens have been previously reported [63]. According to the Lin-Weiss-Bisceglia criteria, adrenal oncocytomas can be benign, borderline malignant potential, and malignant lesions [64]. Few malignant metastatic oncocytomas have been described [65]. On CT and MR scans, the lesion appears as a well-demarcated mass with heterogeneous contrast enhancement due to degenerative changes [65]. Fibrous encapsulation is a typical feature of oncocytoma, having been observed in both benign and malignant variants [65]. Unfortunately, the differentiation from cortical carcinoma is still challenging, similar to that observed for renal oncocytomas and clear cell renal carcinoma [49, 66].

### Malignant adrenal tumors

#### Cortical carcinoma

Cortical carcinoma is an uncommon malignant adrenal tumor, with a bimodal peak of incidence in childhood and middle age [66]. For unclear reasons, it may be functioning especially in females, who also show more frequently an association with endocrine syndromes including Li-Fraumeni syndrome, Beckwith-Wiedemann syndrome, Carney complex, congenital adrenal hyperplasia, and MEN-1 [66, 67]. Non-functioning lesions are asymptomatic as long as they become so large to determine compression and dislocation of adjacent structures.

At US, this lesion presents as a large mass, generally larger than 6 cm in size. Small lesions may show homogeneous echotexture, whereas larger lesions tend to be more heterogeneous due to necrosis or hemorrhage [13]. Intra-lesional echogenic foci due to calcifications can be also observed. At Color Doppler, this lesion appears as a hypervascular mass, which may demonstrate an afferent blood vessel [13]. CEUS reveals a hypervascularized lesion with early enhancement similar to that of pheochromocytoma [14].

On CT, adrenal carcinoma shows a heterogeneous appearance due to necrosis, calcifications, and hemorrhage. After intravenous contrast injection, it demonstrates heterogeneous and mainly peripheral enhancement [66]. As previously reported, the RPW of the carcinoma tends to be less than 40% [67]. Invasion of adjacent structures such as kidney, inferior vena cava, and splenic vessels are common as well as liver metastasis and retroperitoneal lymph nodal locations.

**Fig. 8:**
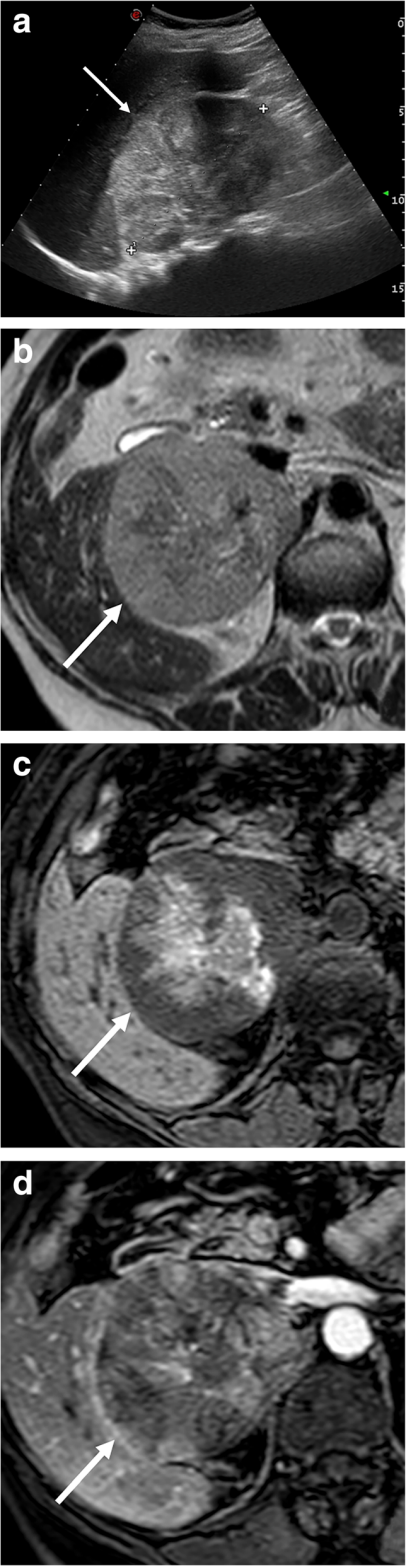
Right cortical carcinoma in a 70-year-old woman admitted to the emergency department for atraumatic abdominal pain. US (**a**) shows a large heterogeneous right adrenal mass (arrow). Axial T2-weighted spin echo (**b**), three-dimensional fat-suppressed T1-weighted gradient recalled echo images before contrast injection (**c**), in arterial phase (**d**) images confirm the presence of a large right adrenal mass with necrotic and hemorrhagic components which present as areas of high signal on unenhanced T1-weighted (**c**) images and non-enhancing areas after contrast injection (**d**)

On MR, cortical carcinoma shows low signal intensity on T1-weighted images, high signal on T2-weighted images, and strong and heterogeneous contrast enhancement with slow washout [68, 69]. Hemorrhagic areas may present as intra-lesional areas of high signal intensity on T1-weighted images (Fig. 8) [69]. Rarely, focal loss of signal intensity on out-of-phase images due to foci of intracytoplasmic fat may be observed [69].

Despite ^18^F-FDG-PET/CT not completely included in the management algorithms of adrenal malignancies, the potential value of this method has been proposed as a second-line test in ruling out suspected recurrences found by CT [70]. Furthermore, a recent study by Cistaro et al. focused on the comparison of ^18^F-FDG-PET/CT with conventional imaging, showing better accuracy of ^18^F-FDG-PET/CT (93.4%) in comparison to CECT (75%) [71]. Nevertheless, further studies will be necessary to confirm these results and its role in the clinical setting.

#### Lymphoma

**Fig. 9:**
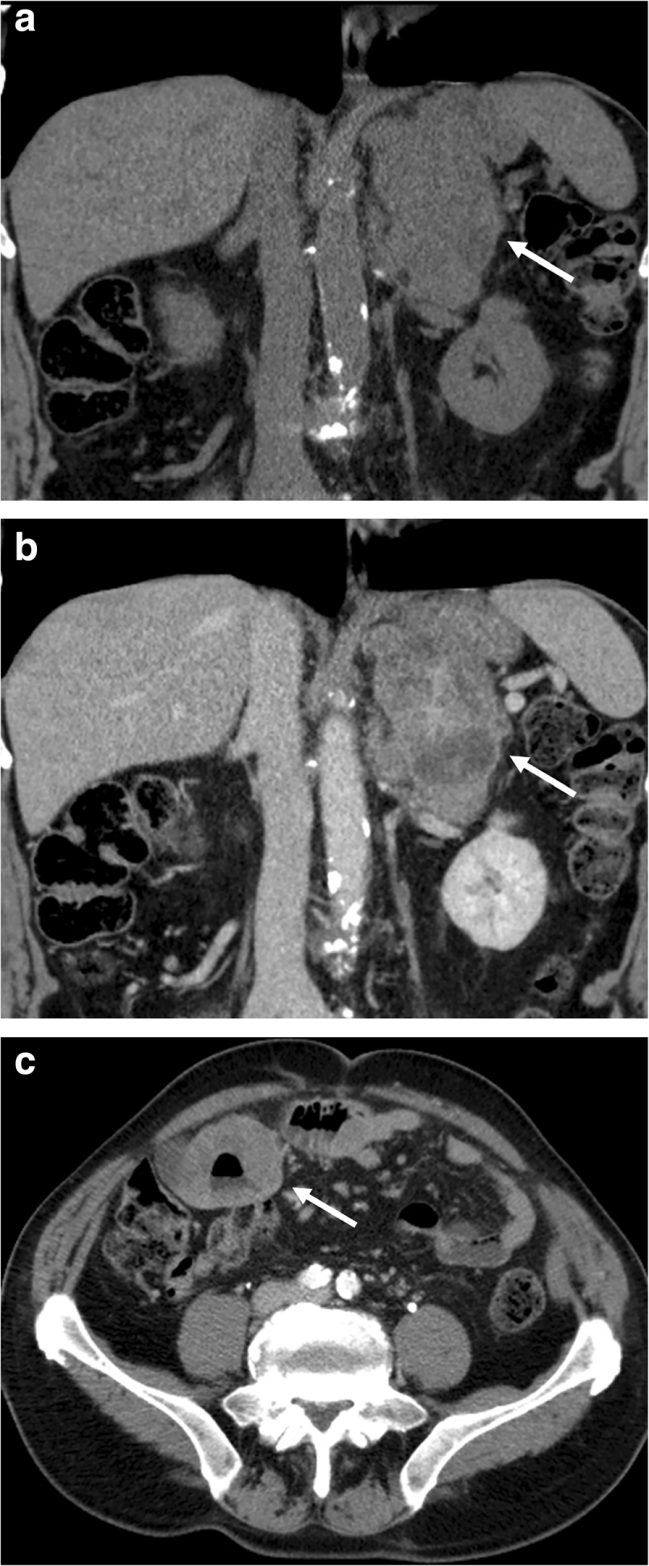
Left adrenal lymphomatous lesion in a 70-year-old man with non-Hodgkin lymphoma. Coronal reformatted CT images before (**a**) and after contrast injection in portal phase (**b**) show a large left adrenal mass (arrow) with inhomogeneous enhancement. Axial CT image of the lower abdomen in portal phase (**c**; arrow) shows the gastrointestinal involvement by lymphoma as aneurysmal dilatation of some loops of the small bowel

The primary adrenal lymphoma is very rare, with the secondary adrenal involvement being more frequent [72]. In 50% of cases, a bilateral location is observed, generally associated with retroperitoneal pathologic lymph nodes [72]. On US, lymphoma appears as hypoechoic with a homogenous or heterogeneous appearance [73]. On CT, primary adrenal lymphoma appears as a single complex hypoattenuating mass, without intra-lesional calcifications, showing slight contrast enhancement (Fig. 9) [74]. In secondary adrenal lymphoma, the adrenals appear enlarged but maintain normal morphology also being more homogeneous than primary lesions. On CECT, secondary lymphomas show slight contrast enhancement and slow washout [72]. At MR, adrenal lymphoma is hypo/isointense on T1-weighted and slightly hyperintense on T2-weighted images [72]. On DWI, lymphomatous lesions always show a restricted pattern of diffusion due to their hypercellularity and high nuclear-to-cytoplasmic ratio, leading to decreased diffusivity of water molecules [75–78]. Nevertheless, this imaging feature is not specific for adrenal lymphoma, but detectable in all locations [79].

#### Metastases

Metastases are the most frequent malignant adrenal lesions. The most common primary tumor metastasizing to the adrenal glands is lung cancer followed by breast, colon, melanoma, kidney, and hepatocellular carcinoma [80]. Bilateral metastases are more common than unilateral metastasis [80]. Metastases are clinically silent, but in some cases extensive involvement of both glands can determine adrenal insufficiency. CT is the method of choice in oncologic patients, while US is used predominantly in pediatric patients. MR is a second-instance method, useful only for further characterization of adrenal lesions. ^18^F-FDG-PET/CT can be helpful to differentiate fat poor adenoma from small metastasis [81].

**Fig. 10:**
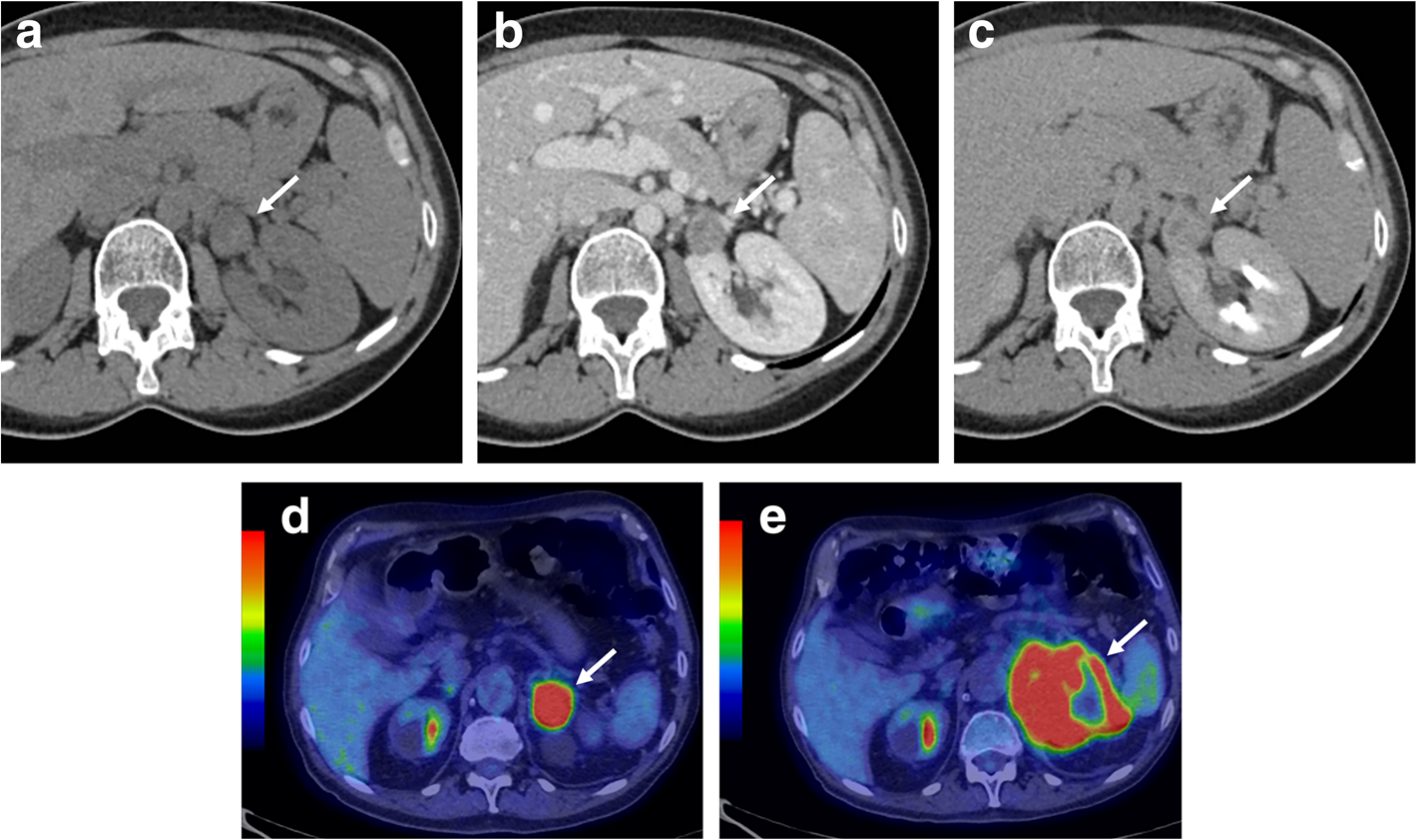
Left adrenal metastasis in a 62-year-old woman with lung cancer. Unenhanced CT (**a**), portal (**b**), and late phase (**c**) images show a left adrenal mass (arrow) with attenuation value of 26 HU on unenhanced CT (**a**) and “absolute percentage washout” of 15%. Staging ^18^F-FDG PET/CT (**d**) performed 3 months later revealed a left adrenal lesion (arrow) with high metabolic activity (SUV_max_ 14 g/mL) and increased in size in comparison with CT images. After chemotherapy, restaging ^18^F-FDG-PET/CT (**e**) showed morpho-functional disease progression of the adrenal lesion (arrow) with signs of central necrosis (SUV_max_ 19 g/mL with central necrotic area of absence of ^18^F-FDG uptake). *Images of* ^*18*^*F-FDG-PET/CT provided by database of Nuclear Medicine Service, Fondazione Istituto G. Giglio, Cefalù, Italy*

**Fig. 11:**
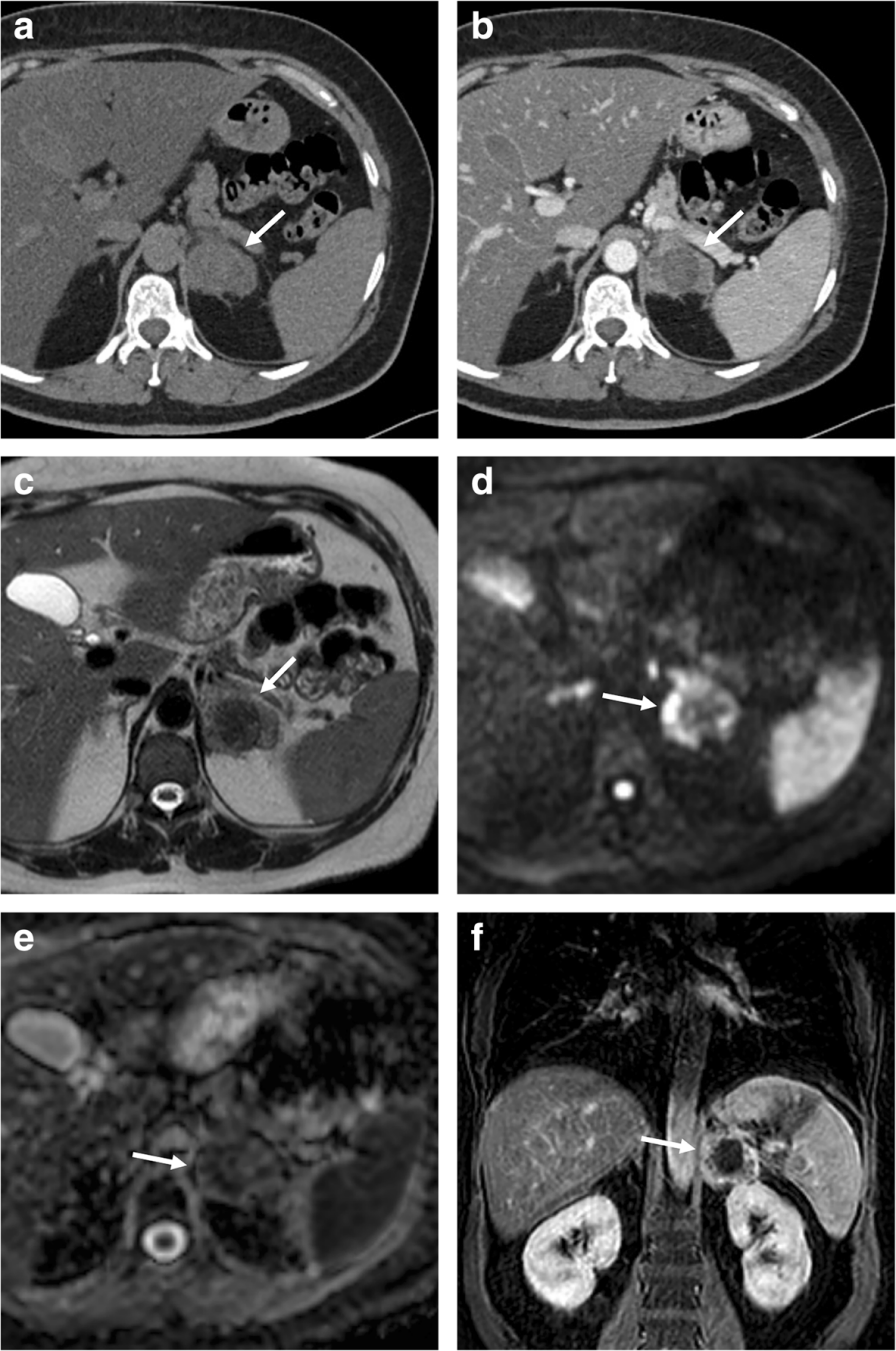
Left adrenal metastasis in a 58-year-old woman with breast cancer. Axial unenhanced CT (**a**), portal phase (**b**), T2-weighted fast spin echo (**c**), b800 s/mm^2^ diffusion-weighted image (**d**), ADC map (**e**), and coronal three-dimensional fat-suppressed T1-weighted gradient recalled echo image in portal phase after contrast injection (**f**) show a left adrenal mass (arrow). This lesion intra-lesional hemorrhage presenting as spontaneously hyperdense area on unenhanced CT (**a**), hypointense on T2-weighted (**c**), and no enhancement on CT (**b**) and MR (**f**) images after contrast injection. The peripheral solid component of the lesion shows also restricted pattern of diffusion (**d**, **e**)

At CT, adrenal metastases appear as focal masses with strong and prolonged enhancement on the portal venous phase, usually more than 120 HU, but slower washout than adenomas (Fig. 10) [21]. At MR, imaging features depend on the type of primary tumor, but generally appear hypointense on T1-weighted and moderately hyperintense on T2-weighted images. Metastases from melanoma may show high signal intensity on T1-weighted images. Moreover, adrenal metastases may show hypointense areas on T2-weighted images with no enhancement after contrast injection due to hemorrhagic intra-lesional components (Fig. 11). Another important imaging feature, which may be helpful to differentiate metastases from adenomas, is the lack of signal loss on out-of-phase images [51]. Conversely, the differential diagnosis between metastasis and primary carcinoma is very difficult, as well as in other districts, with carcinoma generally showing higher local invasiveness [82].

As a functional imaging modality, which provides glucose metabolic information on malignant tumors, ^18^F-FDG-PET/CT has shown encouraging results for the detection of adrenal metastases. Recently, a meta-analysis of Wu et al. evaluating 707 lung cancer patients with 810 adrenal masses has reported an excellent diagnostic performance of ^18^F-FDG-PET/CT supporting its clinical value in the differentiation of adrenal metastases from benign masses [83]. A retrospective study conducted by Kim JY et al. on 325 patients evaluated a combination of morpho-functional parameters using PET/CT demonstrating that unenhanced attenuation of > 10 HU at CT imaging and a SUV_max_ ratio of > 2.5 in PET were significantly associated with adrenal metastases [84]. Further, several studies have compared CT with ^8^F-FDG-PET/CT in this setting. The sensitivity of PET remains still lower than CT due to a limited spatial resolution. For this reason, given a superior specificity, ^18^F-FDG-PET/CT could be considered as a second-stage imaging study for evaluation of indeterminate adrenal lesions [85].

#### Neuroblastoma

Neuroblastoma is the second most common neoplasm in childhood after Wilms tumor, being very rare in adults [86]. This lesion usually presents calcification, necrosis, and intra-lesional hemorrhage [86]. Generally, it is totally asymptomatic, becoming clinically evident when it invades or compresses adjacent structures, metastasizes, or determines a paraneoplastic clinical picture [87].

US is the first imaging modality for this lesion in pediatric patients. Neuroblastoma appears as a large lesion with inhomogeneous echotexture, intra-lesional vascularity at color Doppler, and calcifications in some cases [87]. On CT, it is a lobulated mass, with amorphous or mottled calcifications, usually without capsule and with mild contrast enhancement. Intra-lesional calcifications are detected in 80–90% of cases and represent an important finding to differentiate this neoplasm from Wilms tumor [86, 87].

MR has shown to be more accurate than CT in the characterization of these tumors. Neuroblastoma usually shows a variable appearance, with low signal on T1-weighted and high signal intensity on T2-weighted images, demonstrating heterogeneous contrast enhancement [86, 87].

The MIBG scintigraphy for the detection of this neoplasm has shown equal sensitivity to that for pheochromocytoma [88].

## Conclusion

The widespread use of imaging has dramatically increased the detection of incidental adrenal lesions, which are mostly benign and non-functioning adenomas. Nevertheless, differentiating a benign adrenal lesion from a malignant one can be challenging and is critical especially in oncology patients, since it will greatly affect patient management. In this setting, imaging plays a crucial role with several tools, which can be employed in the attempt to characterize adrenal lesions. Radiologists should recall the strengths and weaknesses of imaging modalities to better help clinicians and surgeons in the management of patients, avoiding a misinterpretation of imaging features which frequently overlap between benign and malignant conditions.
